# Haptoglobin HP2-2 genotype, α-thalassaemia and acute seizures in children living in a malaria-endemic area

**DOI:** 10.1016/j.eplepsyres.2008.04.021

**Published:** 2008-10

**Authors:** Richard Idro, Thomas N. Williams, Samson Gwer, Sophie Uyoga, Alex Macharia, Herbert Opi, Sarah Atkinson, Kathryn Maitland, Piet A. Kager, Dominic Kwiatkowski, Brian G.R. Neville, Charles R.J.C. Newton

**Affiliations:** a Centre for Geographic Medicine Research-Coast, Kenya Medical Research Institute, Kilifi, Kenya; b Department of Paediatrics, Mulago Hospital/Makerere University, Kampala, Uganda; c Department of Paediatrics, John Radcliffe Hospital, Oxford, UK; d Department of Paediatrics, Faculty of Medicine and The Wellcome Trust Centre for Tropical Medicine, Imperial College, London, UK; e Department of Infectious Diseases, Tropical Medicine and AIDS, Academic Medical Centre, Amsterdam, The Netherlands; f The Wellcome Trust Centre for Human Genetics, University of Oxford, UK; g Neurosciences Unit, The Wolfson Centre, UCL, Institute of Child Health, UK

**Keywords:** Haptoglobin genotypes, α-Thalassaemia, Acute seizures and children

## Abstract

Polymorphisms of the haptoglobin (HP) gene and deletions in α-globin gene (α-thalassaemia) are common in malaria-endemic Africa. The same region also has high incidence rates for childhood acute seizures. The haptoglobin HP2-2 genotype has been associated with idiopathic generalized epilepsies and altered iron metabolism in children with α-thalassaemia can potentially interfere with neurotransmission and increase the risk of seizures. We investigated the hypothesis that the HP2-2 genotype and the common African α-globin gene deletions are associated with the increased risk of seizures. 288 children aged 3–156 months admitted with acute seizures to Kilifi District Hospital (Kenya), were matched for ethnicity to an equal number of community controls. The proportion of cases (72/288 [25.0%]) and controls (80/288 [27.8%]) with HP2-2 genotype was similar, *p* = 0.499. The allele frequency of HP2 gene in cases (49.3%) and controls (48.6%) was also similar, *p* = 0.814. Similarly, we found no significant difference between the proportion of cases (177/267 [66.3%]) and controls (186/267 [69.7%]) with deletions in α-globin gene (*p* = 0.403). Among cases, HP2-2 polymorphism and deletions in α-globin gene were neither associated with changes in the type, number or duration of seizures nor did they affect outcome. We conclude that the HP2-2 polymorphism and deletions in α-globin gene are not risk factors for acute seizures in children. Future studies should examine other susceptibility genes.

## Introduction

Seizures are a common neurological symptom in sick children admitted to hospital. In parts of malaria-endemic Africa, over 15% of paediatric admissions may have a history of seizures (Obi et al., 1994; Berkley et al., 2003) and acute seizures are a major risk factor for epilepsy later in life (Carter et al., 2004; Ngoungou et al., 2006). Most seizures occur in febrile children and are precipitated by infections (Akpede et al., 1993; Obi et al., 1994; Waruiru et al., 1996) but in some, genetic factors may be important (Obi et al., 1994; Versteeg et al., 2003).

Polymorphisms of the haptoglobin (HP) gene and deletions in the α-globin gene (α-thalassaemia) are very common in malaria-endemic Africa (Williams et al., 2005; Atkinson et al., 2007) and both the HP2-2 polymorphism and α-thalassaemia have been associated with protection against malaria (Williams et al., 2005; Atkinson et al., 2006, 2007; Wambua et al., 2006). The same region also has high incidence rates for childhood acute seizure disorders (Berkley et al., 2003; Idro et al., 2007). It is unclear whether these polymorphisms or deletions and the high incidence of seizures are associated.

Haptoglobin is a protein that binds free haemoglobin in circulation. The gene has three main polymorphisms: HP1-1, HP1-2 and HP2-2. Proteins expressed by this polymorphic gene have grossly different molecular sizes resulting into different rates of diffusion in the brain. In addition, haptoglobin expressed by the HP2-2 genotype has the least haemoglobin binding capacity and studies have associated this genotype with idiopathic generalized epilepsy (Saccucci et al., 2004; Sadrzadeh et al., 2004). The mean plasma haptoglobin level in patients with idiopathic epilepsy is also lower than that in normal population controls (Panter et al., 1985). It has been suggested that, a defective free haemoglobin clearing system after central nervous system micro-hemorrhage events in persons with the HP2-2 genotype may be involved in the development of chronic seizure disorders (Panter et al., 1985; Sadrzadeh et al., 2004).

The relationship between the α-thalassaemias and acute seizures has not been reported. It is postulated that, children with thalassaemia have an increased oxidative stress on neurons and an altered iron metabolism. The change in iron metabolism may interfere with activities of gamma amino butyric acid and the glutamate system (Auvichayapat et al., 2004) and alter the risk for seizures. Only one retrospective study has examined the incidence of seizures in children with β-thalassaemia and the incidence was lower than that in the general population (Auvichayapat et al., 2004).

We hypothesized that compared to children with HP1-1 and HP1-2 genotypes or those without deletions in the α-globin gene, children with HP2-2 genotype or the common African α-globin gene deletion have an increased risk of developing acute seizures. We recruited children hospitalized with acute seizures and compared their HP genotypes and α-thalassaemia types to that of a matched cohort of community controls.

## Methods

### Study participants

The study was conducted in Kilifi District on the coast of Kenya where most residents are collectively known as the Mijikenda, a Bantu grouping of nine tribes, with the Giriama (45%), Chonyi (33%), and Kauma (11%) forming the major groups. It was part of a bigger epidemiological study that investigated the incidence, risk factors and outcome of acute seizures in children in our community presenting to Kilifi District hospital (Idro et al., 2008). In the catchment area of the hospital, a system of continuous demographic surveillance (DSS) is maintained for a population of about 230,000 (Berkley et al., 2005). Cases for the study were children from within the DSS aged 3–156 months, consecutively admitted to the pediatric wards of the hospital with acute seizures. Children with epilepsy (two lifetime episodes of unprovoked seizures) were excluded. Controls were children born in the hospital who had cord blood samples collected and archived, and were frequency matched to cases on the basis of ethnicity. The primary exposure of interest was the haptoglobin HP2-2 genotype. We estimated that a sample size of 248 cases and 248 controls would give 90% power to detect an OR of 2.0 at 5% level of significance given a prevalence of HP2-2 in the community of 25% (Farrer and Cupples, 1998; Atkinson et al., 2007). Ethical approval for the study was granted by the Kenya Medical Research Institute.

### Procedures

The cases received emergency care and resuscitation based on standard guidelines (WHO, 2000). Parents were then invited to participate in the study and consent obtained. The history included the number, duration, and a description of the seizures and a history of previous seizures in the child. Level of consciousness was assessed using the Blantyre Coma Scale (Molyneux et al., 1989). Specific anti-microbial therapy was given for malaria and for bacterial infections. A child was said to have malaria if s/he had malaria parasitaemia and clinical examination and lab tests excluded other diagnoses. At discharge, all patients were assessed for the presence of neurological deficits and those with deficits referred for follow up care.

Venous blood was drawn and plasma from both cases and controls was immediately separated by centrifugation and the cell pellet frozen and stored at −80 °C. DNA was extracted using commercially available kits according to the manufacturers instructions (PUREGENE^®^ DNA extraction kit, GENTRA SYSTEMS, Boston, MA) and haptoglobin and thalassaemia genotyping was performed by PCR as previously described (Chong et al., 2000; Williams et al., 2005; Atkinson et al., 2006).

### Statistical analysis

To investigate the association between acute seizures and these genetic traits, we compared the prevalence of HP2-2 genotypes, α-thalassaemia deletion types and allele frequency in cases and controls using Pearson's chi-square test and the chi-square test for linear trend. In addition, we compared the seizure types (generalized, focal or secondarily generalized) and the median number of seizures in children with the different genotypes with the Wilcoxon Rank-sum Test. We also performed a sub analysis comparing the genotypes of cases with *Plasmodium falciparum* infection.

## Results

### General description

Three hundred and twenty five children, consecutively admitted with acute seizures to Kilifi District hospital, between December 2004 and August 2005 were recruited. Haptoglobin genotyping was successful in 294 (90.5%) of whom, 288 were frequency matched for ethnicity to an equal number of controls from the birth cohort (six cases did not have matching ethnic groups in the birth cohort). In 267 out 288 (92.7%) children, both the haptoglobin genotype and α-thalassaemia type was available and the cases and controls could be matched for ethnicity. The majority of cases, 167 (58.0%) were from the Giriama ethnic group, 67 (23.3%) were Chonyi, 32 (11.1%) Kauma while the remaining 22 cases belonged to the Digo, Duruma, Jibana, Kambe, Rabai or the migrant Luo ethnic groups.

The median (IQR) age of the cases was 25.6 (14.0–41.1) months and 147 (51.0%) were males. The majority, 276 (95.8%), presented with fever and 104 (36.1%) reported previous episodes of seizures. Malaria was the leading primary diagnosis associated with acute seizures and was seen in 186 (64.6%) cases. Other primary diagnoses included respiratory tract infections, otitis media, acute diarrhoea, skin infections or childhood viral fevers in 79 (27.4%), acute bacterial meningitis/septicaemia in 7 (2.4%) and suspected viral meningo-encephalitis in 7 (2.4%).

### Haptoglobin genotype, α-thalassaemia and acute seizures

The haptoglobin gene polymorphisms and α-globin gene deletions were in Hardy–Weinberg equilibrium. We found no significant association between acute seizures and the haptoglobin HP2-2 genotype (Table 1) or between acute seizures and deletions in the α-globin gene (Table 2).

Seizures in children with malaria often exhibit complex features (focal, prolonged or repeated) (Idro et al., 2007, 2008). We therefore examined this subgroup separately and observed no associations between the HP2-2 polymorphism or deletions in the α-globin gene with malaria-related seizures. In addition, there was no association between this polymorphism or deletions and simple febrile seizures in a group of 79 children whose seizures were associated with respiratory tract and ear infections, acute diarrhoea or skin infections. The proportions of the HP2-2 genotypes in this group of 79 children were: HP1-1 −26.6%, HP1-2 −50.6% and HP2-2 −22.8%; (*χ*^2^ for linear trend compared to the control population 0.011, *p* = 0.915) and that for deletions in the α-globin gene were: no deletion −28.1%, heterozygous deletion −46.9% and homozygous deletion −25.0% (*χ*^2^ for linear trend compared with the control population 1.265, *p* = 0.261).

A parental description of the seizure event was available in 278/288 cases; seizures did not occur in the presence of the consenting parent in 10 cases. Two hundred and thirty four children (84.2%) had generalized seizures, 37 (13.3%) had focal seizures while 7 (2.5%) had secondarily generalized seizures. HP2-2 genotype and deletions in the α-globin gene were not associated with any particular seizure manifestation (focal, generalized or focal with secondary generalization), number of seizures, presentation with status epilepticus or with impairment of consciousness.

## Discussion

Increasingly, genetic factors are being recognised as important risk factors for both acute (febrile seizures, reviewed in (Audenaert et al., 2006)) and chronic (epilepsies, reviewed in (Berkovic et al., 2006)) seizure disorders. Studies have demonstrated an association between the haptoglobin HP2-2 polymorphism and idiopathic generalized epilepsies (Saccucci et al., 2004; Sadrzadeh et al., 2004). In this study we investigated whether this risk extends to acute seizures and whether another common genetic trait in Africa – a 3.7-kDa deletion in the α-globin gene – is associated with the high incidence of acute seizures in this region. The findings do not support our hypothesis: the prevalence of the HP2-2 genotype and α-thalassaemia genotypes were similar in cases and controls; the median number of seizures during the current illness and the frequency of past seizures in children with these polymorphisms/deletions were similar to that in cases without.

Accumulation of iron and iron containing products such as haemoglobin in the interstitial tissues of the brain can initiate and enhance the generation of reactive oxygen species and cause neuronal damage by peroxidation of cell membrane lipids (Panter et al., 1985; Suzer et al., 2000). Haptoglobin is an acute phase protein, which binds free haemoglobin released into circulation following haemolysis. Experimental evidence suggests that hypo-haptoglobinaemia is associated with poor clearance of free haemoglobin from the central nervous system and may lead to seizure disorders (Panter et al., 1985). It is postulated that the inefficient clearance of free haemoglobin in patients with HP2-2 genotype increases unbound free haemoglobin in the brain (and other tissues) and may be associated with increased haemoglobin mediated oxidant stress on cell membranes, oxidative neuronal damage and an increased risk of seizures. Deletions in the α-globin gene may affect a child's susceptibility to acute seizures due to possible changes in neurotransmitter metabolism. Our study, which is based on an even larger number of subjects than previous studies in people with epilepsy suggests that, unlike the idiopathic generalized epilepsies in which the HP2-2 genotype may be one of many contributory genes involved in the polygenic inheritance of the seizure disorder (Saccucci et al., 2004; Sadrzadeh et al., 2004), this polymorphism is not a risk factor for acute seizure disorders. This result is consistent for febrile seizures with both simple and complex characteristics. Although we did not measure plasma haptoglobin levels to directly correlate genotype with phenotype, the evidence for the lack of association between HP2-2 genotype and acute seizures is compelling. It may be possible that there is a time lag between the onset of free haemoglobin induced “oxidative neuronal damage” and manifestation of the seizure disorder, i.e. epilepsy. Alternatively, repeated episodes of “neuronal oxidative damage” are necessary before the seizure disorder can develop.

Despite this finding, family studies in children from this community do provide strong evidence to support a genetic risk factor for acute seizures (Versteeg et al., 2003). It is likely that there are other susceptibility genes. Mutations and single nucleotide polymorphisms in voltage and ligand gated channels such as those described in children with febrile seizures or generalized epilepsy with febrile seizures plus may be possible candidates (Scheffer and Berkovic, 1997; Baulac et al., 2001; Ito et al., 2002; Wallace et al., 2002). Screening for these genetic disorders may be a useful step.

In the study, clinical assessment was used to define seizure type without any EEG data limiting the accuracy of seizure categorization. Bias could have been introduced by only including hospitalized patients (probably a more severely ill group) and selecting controls from a birth cohort born in the same hospital. In addition, our sample size was based on a relatively large OR of 2.0 at 90% power and 5% level of significance. At 80% power and the same level of significance, the sample we recruited could detect a significant result with an OR of 1.8. Other limitations include the limited power for subgroup analysis. These limitations could have biased the results toward the null hypothesis.

In conclusion, the haptoglobin HP2-2 genotype and deletions in the α-globin gene are not risk factors for acute seizures in Kenyan children with and without falciparum malaria infection. Future studies should examine other susceptibility genes.

## Competing interests

We have no competing interests to declare. Apart from funding, the Wellcome Trust and the European Union network 6 BioMalpar consortium have had no role in the conduct of the study, data analysis or the decision to submit the manuscript.

## Figures and Tables

**Table 1:** Haptoglobin genotypes in children with acute seizures and controls

Subject group	Haptoglobin genotypes			Total, *N*	Allele frequency		Total, *N*
	HP1-1, *n* (%)	HP1-2, *N* (%)	HP2-2, *n* (%)		HP1, *n* (%)	HP2, *n* (%)	
Cases	76 (26.4)	140 (48.6)	72 (25.0)	288	292 (50.7)	284 (49.3)	576
Controls	88 (30.5)	120 (41.7)	80 (27.8)	288	296 (51.4)	280 (48.6)	576

The allele frequency of the HP2-2 genotype in cases and controls was similar (*χ*^2^ = 0.57, *p* = 0.499).

**Table 2:** α-Thalassaemia genotypes and acute seizures in children

Subject group	α^−3.7^ ^kDa^ globin deletion types			Total, *N*	Allele frequency		Total, *N*
	No deletion, *n* (%)	Heterozygous, *n* (%)	Homozygous, *n* (%)		No deletion, *n* (%)	Any deletion, *n* (%)	
Cases	90 (33.7)	128 (47.9)	49 (18.4)	267	308 (57.7)	226 (42.3)	534
Controls	81 (30.3)	150 (56.2)	36 (13.5)	267	314 (58.4)	222 (41.6)	534

The proportion of cases and controls with any deletion in the α-globin gene was similar (*χ*^2^ = 0.70, *p* = 0.403).
